# Biochemical characterization of functional domains of the chaperone Cosmc

**DOI:** 10.1371/journal.pone.0180242

**Published:** 2017-06-30

**Authors:** Melinda S. Hanes, Kelley W. Moremen, Richard D. Cummings

**Affiliations:** 1 Department of Surgery, Beth Israel Deaconess Medical Center, Harvard Medical School, Boston, Massachusetts, United States of America; 2 Complex Carbohydrate Research Center, University of Georgia, Athens, Georgia, United States of America; Russian Academy of Medical Sciences, RUSSIAN FEDERATION

## Abstract

Cosmc is an endoplasmic reticulum chaperone necessary for normal protein O-GalNAc glycosylation through regulation of T-synthase, its single client. Loss-of-function of Cosmc results in expression of the Tn antigen, which is associated with multiple human diseases including cancer. Despite intense interest in dysregulated expression of the Tn antigen, little is known about the structure and function of Cosmc, including domain organization, secondary structure, oligomerization, and co-factors. Limited proteolysis experiments show that Cosmc contains a structured N-terminal domain (CosmcΔ256), and biochemical characterization of CosmcΔ256 reveals wild type chaperone activity. Interestingly, CosmcE152K, which shows loss of function *in vivo*, exhibits wild type-like activity *in vitro*. Cosmc and CosmcE152K heterogeneously oligomerize and form monomeric, dimeric, trimeric, and tetrameric species, while CosmcΔ256 is predominantly monomeric as characterized by chemical crosslinking and blue native page electrophoresis. Additionally, Cosmc selectively binds divalent cations in thermal shift assays and metal binding is abrogated by the CosmcΔ256 truncation, and perturbed by the E152K mutation. Therefore, the N-terminal domain of Cosmc mediates T-synthase binding and chaperone function, whereas the C-terminal domain is necessary for oligomerization and metal binding. Our results provide new structure-function insight to Cosmc, indicate that Cosmc behaves as a modular protein and suggests points of modulation or regulation of *in vivo* chaperone function.

## Introduction

Cosmc is an endoplasmic reticulum (ER) chaperone that is required for the activity of T-synthase, an essential glycosyltransferase (GT), and its only known client [1]. T-synthase (Core 1 β1−3 galactosyltransferase–C1GALT1) is necessary for normal O-glycoprotein biogenesis and elongates the Tn antigen (GalNAcα1-Ser/Thr glycopeptide) by the addition of galactose to form the core 1 glycan structure (Galβ1−3GalNAcα1-Ser/Thr) [2]. This type of O-GalNAc glycosylation is a ubiquitous and complex posttranslational modification, where ~80% of proteins that enter the secretory pathway are predicted to contain one or more O-GalNAc modifications, which are temporally and spatially regulated [3, 4]. Although *T-synthase* is found throughout the animal kingdom, vertebrates require *Cosmc (C1GALT1C1)* and in cells that lack either protein, abnormal O-glycoproteins accumulate that express the Tn antigen [5–7]. Tn is rarely exposed in normal O-GalNAc glycoproteins and not typically detectable in healthy tissues.

Cosmc has a pivotal role in regulating Tn expression *in vivo*, and defects in Cosmc expression and function directly lead to abnormal protein O-glycosylation, causing a pleiotropic effect on the cellular glycoproteome. Genetic defects in *Cosmc* have repeatedly been identified as causal for Tn expression in human and mouse cell lines, and polymorphisms in *Cosmc* have been identified as a key marker for inflammatory bowel disease [1, 8–11]. Despite its central role in regulating Tn, the structure and biochemical mechanism of the interaction of Cosmc with T-synthase are unclear.

**Fig 1:**
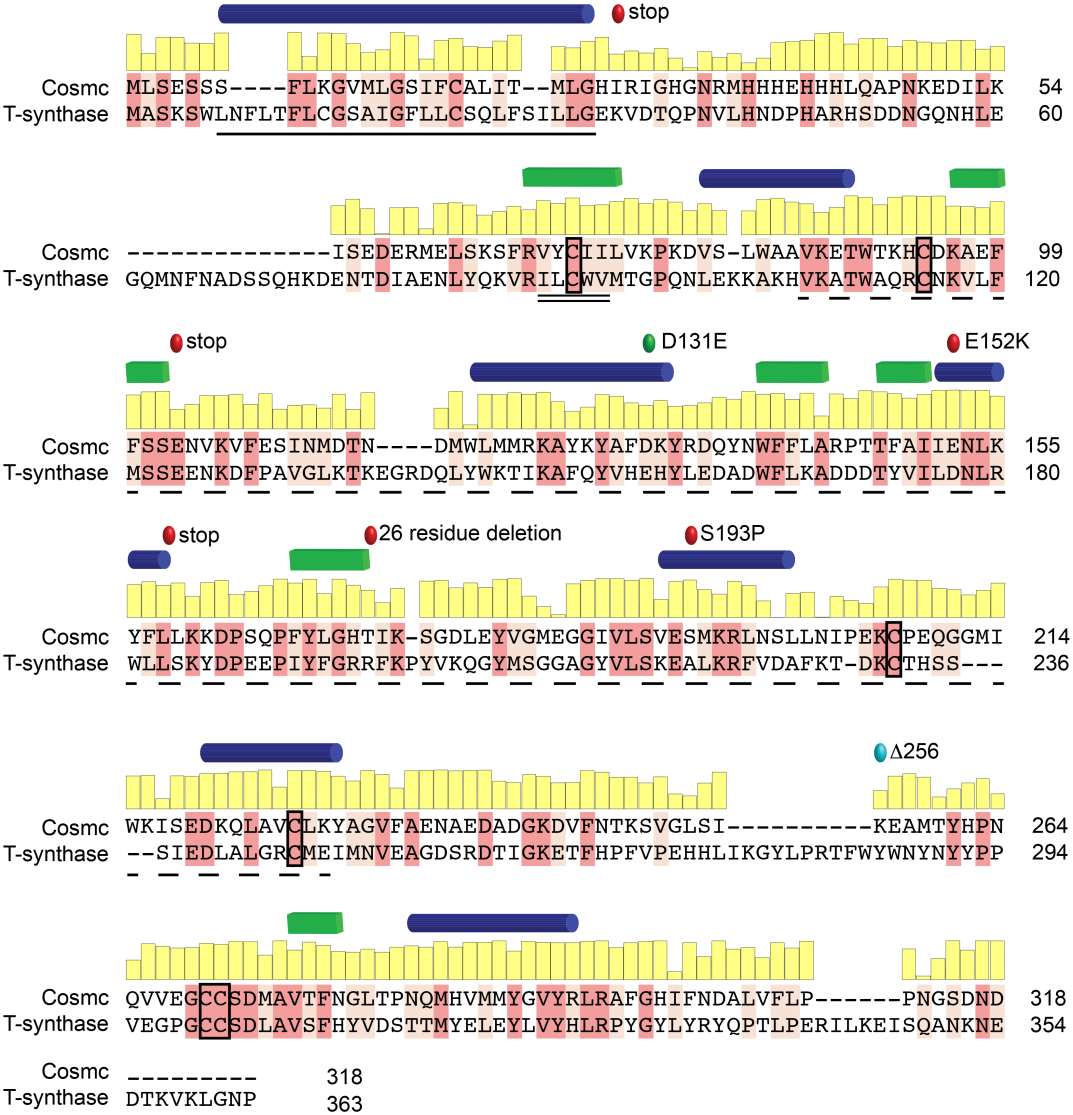
Sequence alignment between Cosmc and T-synthase. A pairwise sequence alignment between human Cosmc and its client, human T-synthase, highlighting identical (dark pink) and conserved residues (light pink). The conservation at each residue position in Cosmc is shown in a yellow bar graph above the sequence. The putative transmembrane domain of Cosmc (black line), the Cosmc recognition region in T-synthase (black double line), and the galactosyltransferase domain of T-synthase (black dashed line) are shown. Additionally, inactivating point mutations, E152K, and S193P, and premature stop codons (red circles), along with a permissive substitution, D131E, (green circle) are indicated. The designed truncation mutation CosmcΔ256 is also annotated (light blue circle). Predicted secondary structure elements, helices (blue) and strands (green), are shown for Cosmc above the sequence [14]; conservation was calculated from a multiple sequence alignment of over 100 Cosmc orthologs using ConSurf [15].

Structural information is used to predict active sites and functional residues, and guides mutagenesis work. However, such information for Cosmc is unavailable, and even low resolution structural information, *e*.*g*. secondary structure, protein fold, domain architecture, cofactor binding, and oligomerization behavior, has yet to be described. Furthermore, because Cosmc shares little sequence homology to any known protein structure, a reliable homology model is challenging. However, Cosmc shares significant (27%) sequence identity with its client, T-synthase, which is a member of the CAZy GT31 family (Fig 1). This homology makes Cosmc and T-synthase unique among chaperone-client pairs, and additionally may provide clues to the structure of Cosmc. Furthermore, the client binding site on Cosmc is unknown, and mutational data is largely lacking, save for the *Cosmc* mutations E152K and S193P, which are nonfunctional *in vivo*, and another study indicating that two of the six cysteine residues in Cosmc are essential *in vitro* [9, 12, 13].

Cofactors, such as ATP, Ca^2+^ or Zn^2+^, are often important in chaperone cycles (for BiP(GRP78), calnexin, and calreticulin), however no cofactors have yet been identified for Cosmc. In fact, our earlier work showed that though Cosmc has some affinity for ATP, it does not affect *in vitro* chaperone activity [16, 17]. Divalent cations are also necessary for function of most GTs, including T-synthase, though it is unknown whether Cosmc might also binds metals [18]. Oligomerization is another key feature which has been particularly extensively implicated in chaperones like Hsp90 and Hsp70 [19]. For Cosmc, oligomerization has not yet been characterized, with the exception of a known intermolecular disulfide bond within the transmembrane region essential for ER retention [20].

To answer these fundamental and outstanding questions, we used biochemical and biophysical tools to characterize Cosmc. These experiments elucidate the domain organization and attribute functionality to each domain: Cosmc has an N-terminal chaperone domain, and a C-terminal domain that mediates oligomerization and Zn^2+^ binding. These data describing Cosmc structure and function yield valuable insights into mechanisms of Cosmc function.

## Results

### Optimizing recombinant expression and purification of Cosmc

**Fig 2:**
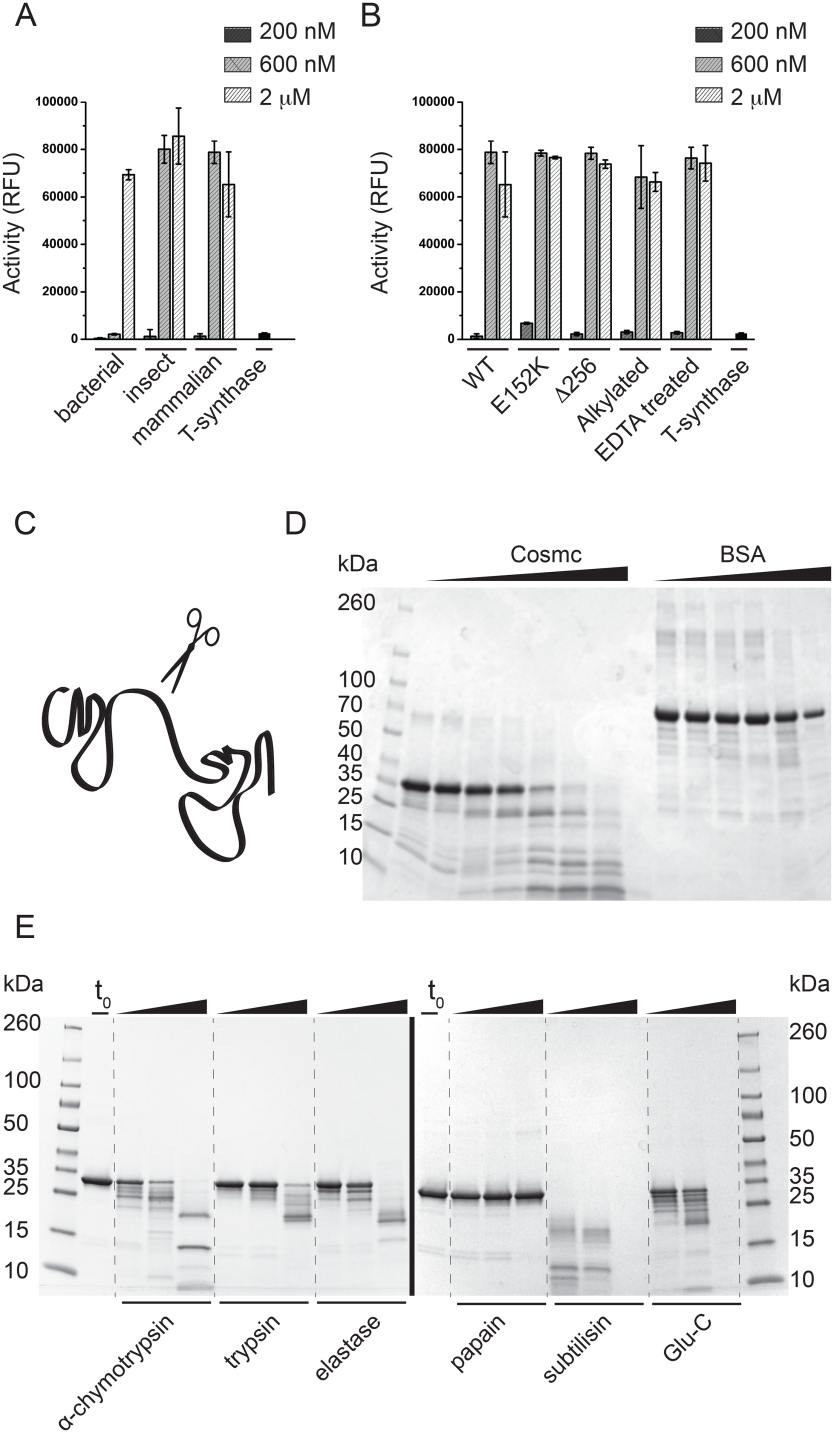
Cosmc functional assays and limited proteolysis experiments. **(A)** Active Cosmc can be recombinantly expressed and purified from bacterial, insect, and mammalian host cells. Cosmc was tested in a chaperone assay at three concentrations, 200nM, 600nM and 2uM. Data is reported as mean and standard deviation from quadruplicate measurements. **(B)** Chaperone activity assay comparing the WT and mutant Cosmc proteins, along with EDTA-treated and alkylated Cosmc. Data is reported as mean and standard deviation for quadruplicate measurements. **(C)** A cartoon representation of a two-domain protein, where unstructured regions at the domain boundaries are more susceptible to protease cleavage. **(D)** Limited proteolysis experiment in which Cosmc was digested with trypsin at 1:500 molar ratio and analyzed by SDS-PAGE (this experiment utilized the bacterially produced Cosmc). Cosmc samples were taken before trypsin addition (lane 2), immediately after trypsin addition (lane 3), and after 5min, 10min, 30min, 1hr, and 3hr (lanes 4–8). For the BSA control, samples were taken before protease addition (lane 9), after 5min, 10min, 30min, 1hr, and 3hr (lanes 10–14). **(E)** Limited proteolysis experiments with Cosmc purified from HEK293F cells using a broad range of proteases (*i-vi*). Samples were taken for SDS-PAGE before protease addition (lane 2), and after 20min, 1hr, and overnight, with α-chymotrypsin, trypsin, elastase, papain, subtilisin, and endoproteinase Glu-C.

Obtaining large amounts of highly purified material is a common bottleneck for biochemical and biophysical studies, and though our previous work relied upon a baculovirus insect cell expression system for expressing recombinant Cosmc, it is not ideal because it is low yield [17, 19, 21]. We therefore pursued a variety of constructs for Cosmc expression in *E*. *coli*, including co-expression with bacterial chaperones, specialized host strains, periplasmic localization, fusion proteins, and low temperature expression. Because Cosmc is a resident of the ER and therefore is expected to contain oxidized cysteine residues, we tested an *E*. *coli* strain (Shuffle^®^, NEB) that promotes disulfide bond formation, and also tested secretion signals for periplasmic localization. Despite these substantial efforts, expression of soluble Cosmc was not significantly improved in *E*. *coli*. However, although Cosmc is poorly soluble, it is highly expressed in the insoluble, or inclusion body fraction. We found that we could purify Cosmc from the insoluble fraction and refold it into a functional chaperone (Fig 2A). Although high protein yields can be obtained from refolding protein preparations, it is laborious and not ideal for biochemical and biophysical studies. Therefore, we relied upon a robust mammalian expression system using HEK293F suspension cells similar to that reported by Moremen and coworkers for the majority of studies reported herein [22]. Previously we reported that the single N-glycosylation sequon at position 313 is not well utilized in mammalian cells [16]. In agreement with this, N-glycosidase treatment of recombinant Cosmc did not produce a gel shift, consistent with a lack of N-glycans (S1 Fig).

Our *in vitro* chaperone assay for Cosmc function relies on the enzymatic activity of the client, T-synthase, as a reporter [17]. Recombinant T-synthase subjected to multiple freeze thaw cycles shows reduced galactosyltransferase activity, which is regained upon addition of Cosmc. Importantly, Cosmc, but not other control proteins including the ER chaperone Grp78/BiP, galectin-3, or BSA, have *in vitro* chaperone activity against T-synthase [17].

Cosmc produced in all three hosts (insect, bacterial and mammalian cells) is functional in this *in vitro* chaperone assay (Fig 2A). Cosmc purified from *E*. *coli* requires a higher concentration for maximal chaperone activity, likely because only a fraction of the refolded protein may be active. Because refolding preparations may contain heterogeneously folded species, they are often not ideal for biochemical and biophysical studies. However, at the highest concentration used, the chaperone activity of the refolded protein is equal to that of the mammalian and insect produced protein. This new information indicates that no posttranslational modifications of Cosmc, as would be generated in mammalian cells, are required for *in vitro* activity.

### Limited proteolysis of Cosmc generates a stable and functional N-terminal core domain

Domain architecture is a key component of protein modularity, and independent domains in a protein may have a unique functional role. Limited digestion with protease can reveal protein domain structures, as regions of high proteolytic susceptibility correspond to disordered regions, and conversely resistance corresponds to structural order (Fig 2C) [23–25]. Limited proteolysis of the recombinant Cosmc produced in *E*. *coli* reveals a trypsin resistant fragment approximately 25 kDa in mass, unlike that seen in the degradation of BSA, which is not stepwise (Fig 2D). Although BSA is a multi-domain protein, the domains interact significantly and do not behave independently. The trypsin-resistant fragment of Cosmc was excised from SDS-PAGE and the site of cleavage was identified as Lysine-256 by high-resolution LC-MS/MS at the Emory University School of Medicine Proteomics Core. Because these initial studies were performed using protein produced in *E*. *coli* cells, we also characterized the proteolysis pattern of the WT Cosmc produced in mammalian cells. Cosmc purified from mammalian cells is also degraded in a stepwise fashion by a range of proteases, consistent with the presence of a structured core domain, which is relatively independent from a second, smaller, more disordered C-terminal domain (Fig 2E). We recombinantly expressed CosmcΔ256 a truncation mutant designed based on our proteolysis results in which the C-terminal 62 residues are deleted. CosmcΔ256 is a well expressed, folded, and soluble protein, which is in line with the proteolysis results suggesting that it is a relatively independent domain. Again, because the truncation mutant was experimentally identified based on the *E*. *coli* produced protein, we performed a direct comparison between recombinant CosmcΔ256 from mammalian cells, and mammalian and bacterially produced WT Cosmc, which show similar domain structures (S2 Fig).

To test whether the C-terminal residues are required for *in vitro* chaperone function, we characterized CosmcΔ256 in an *in vitro* chaperone assay of T-synthase activity along with the point mutant CosmcE152K, which was identified as a loss of function mutant in patients with Tn syndrome (Fig 2B). CosmcE152K arises from a single nucleotide mutation at base 454 in a patient with Tn syndrome, and although it is expressed at a similar level to WT, the point mutation results in a complete loss of T-synthase function in cells [13]. Both CosmcΔ256 and Cosmc E152K are fully functional *in vitro* chaperones. Additionally, we tested the chaperone activity of Cosmc after reducing and alkylating the protein, and of Cosmc after treatment with EDTA, and these results showed similar activity to the untreated protein. These experiments were conducted to characterize whether the cysteine residues in Cosmc were directly involved in client interactions, and the results agree with our earlier observations that alkylation does not affect *in vitro* chaperone function [19]. Our earlier work suggested that at least one cysteine residue in Cosmc was free, and labeled by N-ethylmaleimide following the mass of the intact protein [19].

### Low-resolution structural characterization of Cosmc by circular dichroism

**Fig 3:**
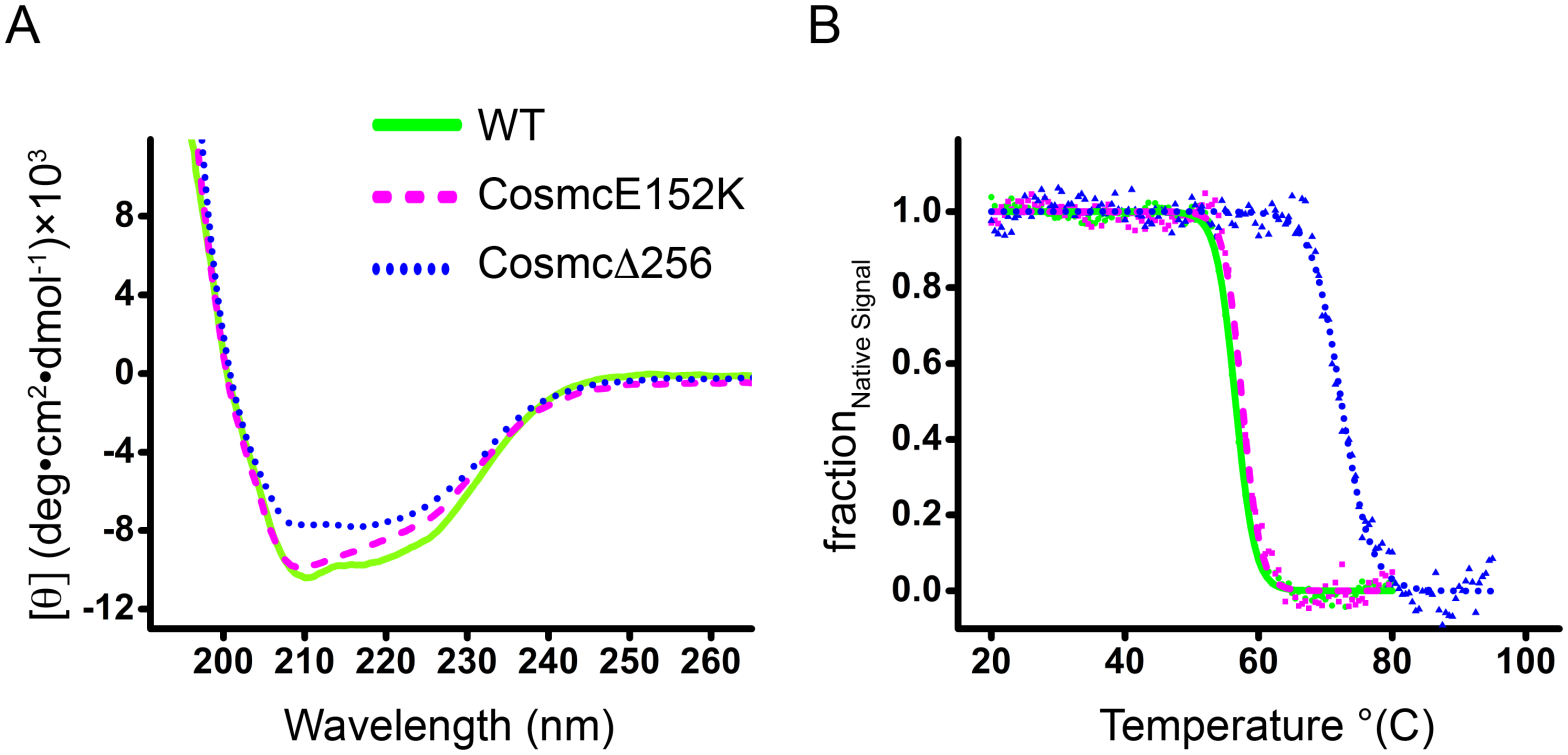
CD spectra and thermal denaturation curves. WT Cosmc (solid line, green), CosmcE152K (dashed line, magenta), and CosmcΔ256 (dotted line, blue) are plotted together for comparison. **(A)** The far UV CD spectra of Cosmc and mutants are indicative of well-folded proteins with significant helical content. **(B)** Thermal denaturation was followed by recording the CD signal at 222 nm with a slope of 1°C/min. Data are presented as fraction of the native signal (*F*_*fold*_) as a function of temperature.

The secondary structure and thermal stability of Cosmc was characterized using circular dichroism (CD) spectroscopy (Fig 3A and 3B). CD is a valuable technique for studying the secondary structure of protein, and is especially useful when high-resolution structural data is lacking. The far-UV CD spectra were collected for Cosmc, CosmcE152K, and CosmcΔ256, and indicate all are well-folded proteins (Fig 3A). Thermal denaturation curves show that Cosmc and CosmcE152K are similarly thermostable, while the truncation CosmcΔ256 is significantly stabilizing (Fig 3B). Spectra were deconvoluted using the K2D2 algorithm on the Dichroweb server to obtain a quantitative estimate of the amount of α-helix, β-sheet, and coil secondary structure components (Table 1) [26]. Cosmc is comprised of ~30% α-helix and ~10% β-sheet, which is unperturbed by the E152K mutation, or the truncation CosmcΔ256. Cosmc is not significantly related to any protein with a known structure, however T-synthase (by homology) is predicted to adopt a mixed α/β GT-A fold, which may extend to Cosmc [27]. Of the reported GT structures, T-synthase and Cosmc are in CAZy GT31 and are most similar to mouse manic fringe (Mfng), a β-1,3-N-acetylglucosaminyltransferase (24% sequence identity between human T-synthase and Mfng). Although Cosmc is more distantly related to Mfng (13% sequence identity), given their shared evolutionary history, it is likely that Cosmc and Mfng share a similar tertiary structure. Therefore, for comparison, the secondary structure composition of Mfng is included in Table 1 [28]. The calculated percentages of α/β secondary structure in Cosmc that result from the experimental CD spectra are in accordance with a GT-A type fold. Note that the GT homology domain within T-synthase corresponds to residues 86–229 of Cosmc, which is entirely contained within the truncation mutant, CosmcΔ256.

**Table 1:** CD characterization of Cosmc.

	Secondary Structure			T_m_ (°C)
	α-helix	β-sheet	coil	
Cosmc*	0.30	0.13	0.57	56.5 ± 0.1
Cosmc E152K*	0.31	0.11	0.58	57.6 ± 0.1
CosmcΔ256*	0.31	0.12	0.57	72.4 ± 0.2
Mfng^†^	0.32	0.16	0.52	N.T.

*The fraction of each secondary structure is given for each protein along with the T_m_ value. Secondary structure deconvolutions were performed from the CD spectra with the DichroWeb K2D2 algorithm [26].

^†^Mfng (Mouse manic fringe), the closest homolog to Cosmc with a known structure, is shown for comparison, and its secondary structure were calculated from its structure (PDB ID 2J0B) with 2Struc using DSSP [29].

### Cosmc oligomerization depends on the C-terminal domain

**Fig 4:**
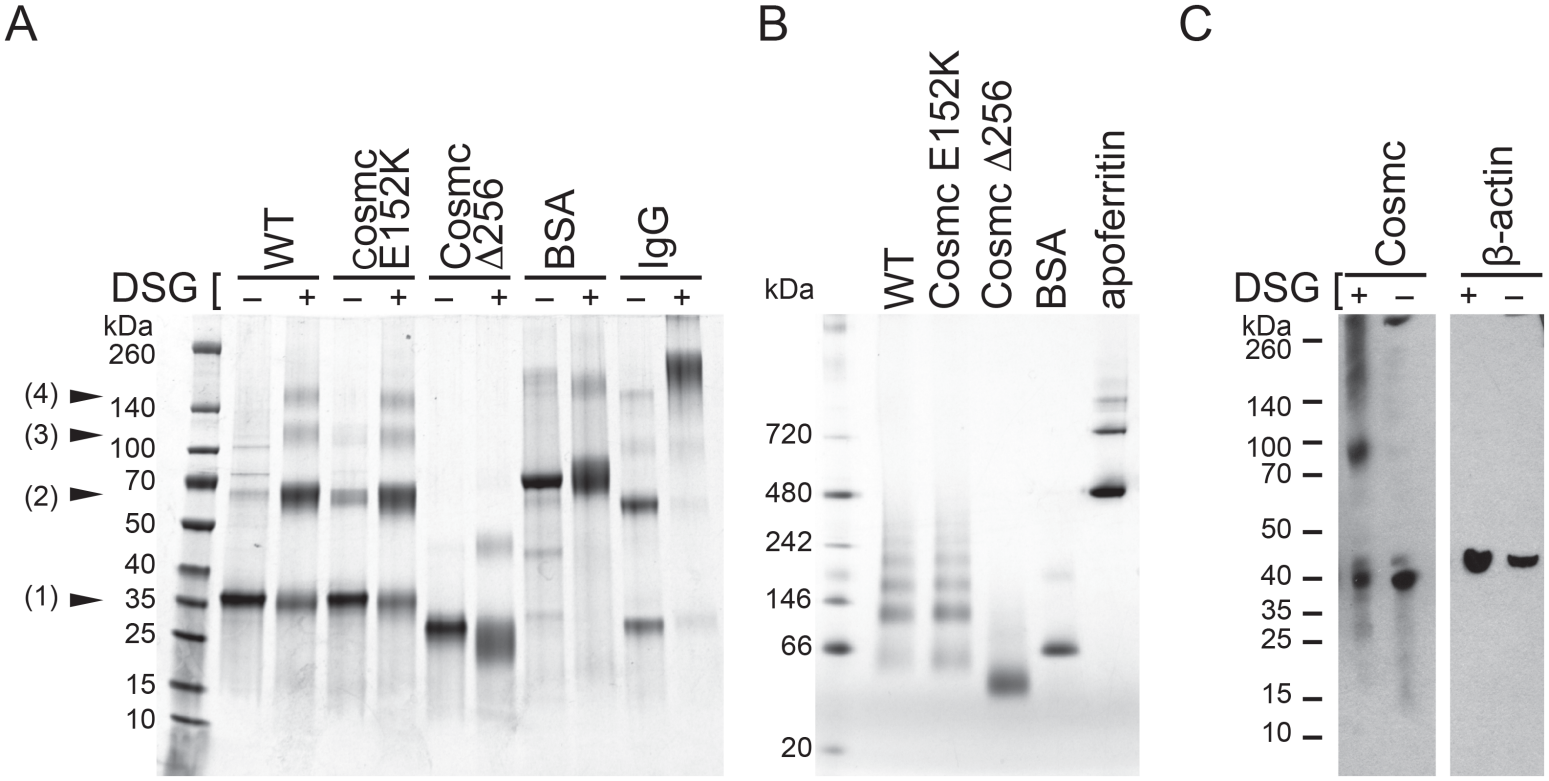
Oligomeric behavior of WT, CosmcE152K, and CosmcΔ256. **(A)** Chemical crosslinking followed by SDS PAGE shows products corresponding to the mass of Cosmc monomer (1), dimer (2), trimer (3), and tetramer (4) for Cosmc and CosmcE152K, while CosmcΔ256 yields only monomer and small amounts of dimer species. Cosmc was treated with either the crosslinker DSG (+), or mock treated (-), along with negative and positive controls, BSA and IgG respectively, for protein oligomerization. **(B**) BN-PAGE analysis of Cosmc along with monomeric and oligomeric control proteins, BSA and apoferritin, respectively. WT and CosmcE152K show heterogeneous oligomers, while CosmcΔ256 shows a single predominant species. **(C)** Chemical crosslinking with DSG of full length Cosmc from crude HEK293SC lysates shows monomeric, dimeric, and trimeric species. Cosmc from cell lysates was detected by western blotting.

Protein oligomerization is often important for biological function. To address whether Cosmc is oligomeric we examined its forms after chemical crosslinking and blue native PAGE (BN-PAGE) (Fig 4A and 4B). After crosslinking, heterogeneous Cosmc products with molecular weights corresponding to monomeric (33.6 kDa), dimeric (67.2 kDa), trimeric (100.8 kDa), and tetrameric (134.4 kDa) species are observed (Fig 4A). Positive and negative controls, IgG, a dimeric protein, and BSA, a monomer, were also included in the experiment, and showed expected results, i.e. IgG was cross-linked whereas BSA was not cross-linked. Next, we exploited the truncation mutant CosmcΔ256 and CosmcE152K to help elucidate the oligomerization determinants within Cosmc. CosmcE52K oligomerizes similarly to WT, while CosmcΔ256 shows significantly reduced oligomerization. In general the degree of crosslinking does not directly report the fraction of any distinct oligomeric species present in solution; thus, the crosslinking data is consistent with the interpretation that Cosmc exists in either a heterogeneous oligomeric population and/or a homogeneous tetrameric population with varying crosslinking efficiencies between subunits. To differentiate between these models, we performed blue native PAGE (BN-PAGE) (Fig 4B). BN-PAGE analysis of Cosmc shows a heterogeneous oligomeric population with apparent masses from 60 kDa to 240 kDa. Apoferritin, an oligomer (24 subunits) with an oligomeric mass of 443 kDa does not produce a laddering effect, nor does BSA, monomeric with mass of 67 kDa, which were included as controls. In agreement with the crosslinking results, CosmcE152K is similar to WT, while CosmcΔ256 shows dramatically reduced oligomerization. These results indicate that the C-terminal domain is required for oligomerization.

### Full length Cosmc oligomerizes in HEK293 native cell lysates

Recombinant protein production can lead to artifacts due to potentially high concentrations, and additionally, the type of construct used. In our case the recombinant Cosmc constructs lack the transmembrane domain present in the native Cosmc, which could alter the oligomerization properties. To determine whether the property of being cross-linked as observed for the recombinant protein is a feature shared by full length Cosmc, we performed the crosslinking experiments on mammalian cell lysates. Full length Cosmc with a C-terminal HPC4 tag was transfected into HEK293 Simple Cells, in which endogenous Cosmc has been silenced. After crosslinking, Cosmc monomers (37.5 kDa) and dimers (75 kDa) are observed as well as indistinct bands of higher molecular mass, whereas a negative control, β-actin, is not crosslinked (Fig 4C). Therefore heterogeneous oligomerization is a feature shared by the full-length protein expressed at more physiologic conditions and not simply a property of the recombinantly purified protein. These results are in contrast to similar crosslinking studies on active T-synthase in cells in which a discrete dimeric species was observed [19].

### Cosmc binds selective divalent cations

Like other glycosyltransferases, T-synthase binds to Mn^2+^, which is coordinated to the sugar donor substrate, UDP-Gal [5, 18]. Because Cosmc shares sequence homology with T-synthase, we evaluated divalent cations for their ability to stabilize Cosmc in a thermal shift assay, to ascertain whether the sequence homology extended to functional homology (Table 2). Thermal shift assays characterize the midpoint of the thermal unfolding transition (*T*_*m*_) in response to addition of potential ligands, which result in an increase in *T*_*m*_ due to preferential interaction with the native relative to the denatured state [30]. The thermal assays utilized the reporter dye, Sypro Orange (Molecular Probes), which binds to the hydrophobic regions exposed upon protein denaturation.

**Table 2:** Selective divalent cations stabilize Cosmc.

	Cosmc ΔT_m_ (°C)	CosmcE152K ΔT_m_ (°C)	CosmcΔ256 ΔT_m_ (°C)
untreated	9.6 ± 0.2	2.3 ± 1.4	1.1 ± 0.5
ZnCl_2_	13.5 ± 0.3	7.5 ± 1.2	-2.2 ± 0.3
FeCl_2_	7.1 ± 0.4	2.9 ± 1.6	-8.8 ± 0.2
MgCl_2_	-0.2 ± 0.5	N.T.	N.T.
CaCl_2_	-0.9 ± 0.3	N.T.	N.T.
MnCl_2_	0.2 ± 0.2	N.T.	N.T.
CdCl_2_	-2.5 ± 0.2	N.T.	N.T.
CoCl_2_	-2.6 ± 0.1	N.T.	N.T.
CuCl_2_	-7.9 ± 0.3	N.T.	N.T.
NiCl_2_	-2.8 ± 0.1	N.T.	N.T.
ATP	-2.5 ± 0.3	N.T.	N.T.
4MU-GalNAc	-1.4 ± 0.4	N.T.	N.T.
UDP-Gal	0.4 ± 0.4	N.T.	N.T.

ΔT_m_ values for divalent cations are calculated with respect to the EDTA treated samples (ΔT_m_ = T_m_ − T_m(EDTA treated)_). Metals that do not stabilize Cosmc were not tested (N.T.) for CosmcE152K and CosmcΔ256. ΔT_m_ values for ATP, 4MU-GalNAc, UDP-Gal are calculated with respect to untreated samples (ΔT_m_ = T_m_ − T_m(untreated)_).

Although T-synthase specifically requires Mn^2+^ for function, Mn^2+^ does not bind to Cosmc [5], however, given the homology between Cosmc and T-synthase, we tested other T-synthase substrates, UDP-Gal and 4MU-α-GalNAc, which also do not interact with Cosmc. Lastly, because in our early work, Cosmc was pulled down by ATP-sepharose resin, we also tested ATP [16]. However in a subsequent publication, we demonstrated that ATP has no effect on *in vitro* chaperone activity, and this is in line with the thermal shift data detecting no interaction between Cosmc and ATP [17].

Cosmc is selectively stabilized by Zn^2+^ and Fe^2+^ from a panel of divalent cations, which included Mg^2+^, Ca^2+^, Mn^2+^, Cd^2+^, Co^2+^, Cu^2+^, and Ni^2+^ (Table 2). We also tested ATP, UDP-Gal, and 4-methylumbelliferyl-α-GalNAc, none of which resulted in a stabilizing *T*_*m*_ shift (Table 2). The two mutants, CosmcE152K and CosmcΔ256 were characterized with the metals that stabilized wild type Cosmc. The truncation mutant is not stabilized by Zn^2+^ and Fe^2+^, but instead is destabilized, while the mutation E152K reduces Zn^2+^ and Fe^2+^ binding. These observations suggest that metal binding is mediated by the C-terminal domain, and with an additional contribution by Cosmc residue E152. The abrogation of metal binding in the truncation demonstrates that it is essential for metal binding, and the reduction in binding by E152K indicates it also has a role. Often cysteine, histidine, aspartate, and glutamate residues are involved in chelating zinc or other metals [31]. To test the potential involvement of the cysteine residues we alkylated the protein; alkylation did not affect its metal binding activity by thermal shift assays. Bioinformatics prediction servers based on protein sequence (ZincPred, MetalPredator, ZincExplorer, MetalDetector v2.0) yielded ambiguous results regarding the residues that might be directly involved in metal chelation.

In order to identify whether endogenous metals are bound in recombinantly purified Cosmc preparations, we analyzed a sample using inductively coupled plasma mass spectrometry (ICP-MS). The sample had not been treated with exogenous metal (or metal chelator). The results indicate the presence of zinc at a concentration equal to ~20% of the total protein concentration. Nickel was also identified in the sample and its presence is likely an artifact arising from Cosmc purification using Ni-NTA resin using the endogenous histidine-rich sequence at the N-terminus of the protein (HHHEHHH). Thus, a fraction of purified Cosmc has detectable zinc, suggesting that Cosmc can occur in a metalloprotein form.

### EDTA treatment destabilizes the oligomeric form of Cosmc

**Fig 5:**
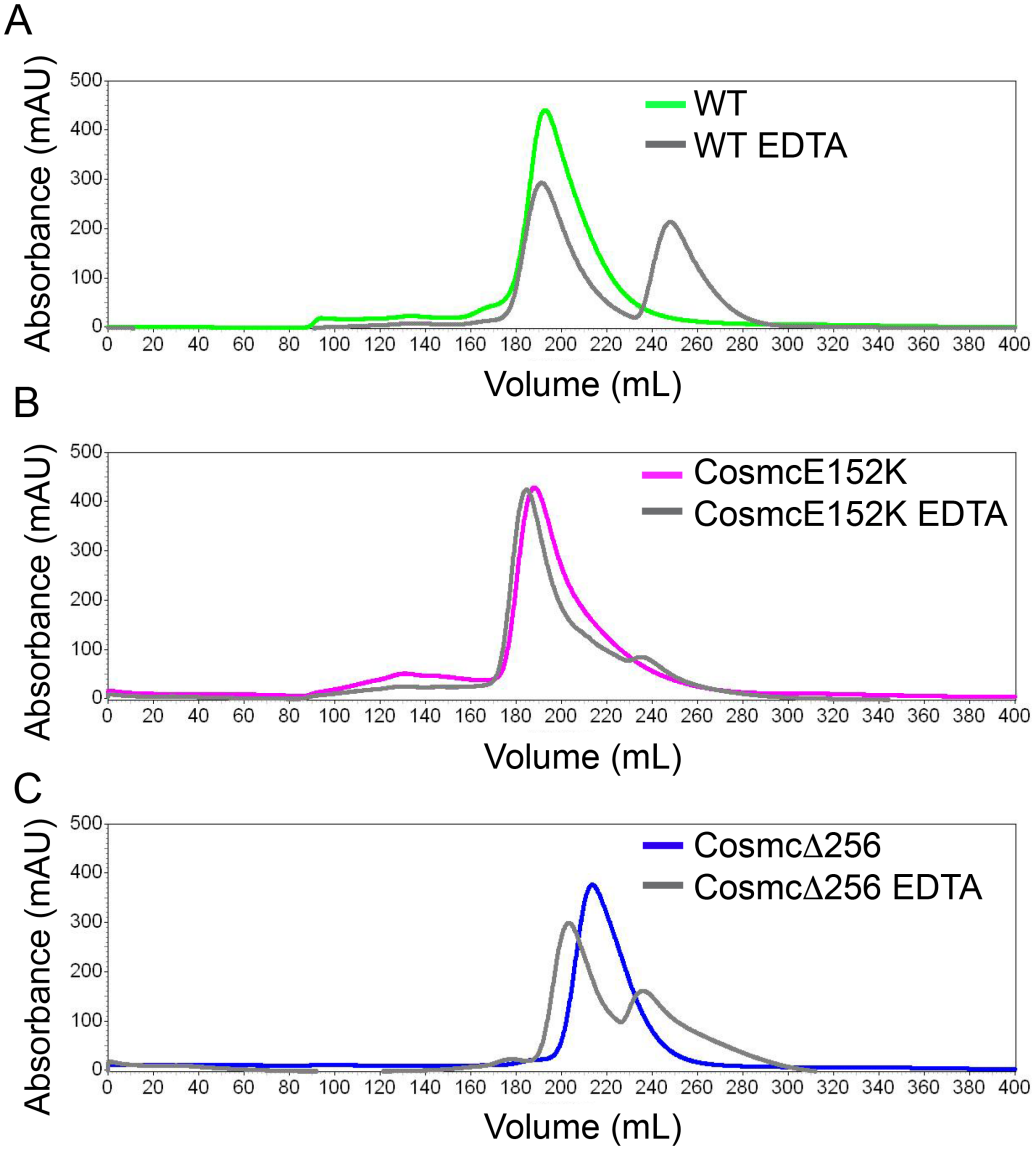
SEC of Cosmc, CosmcE152K, and CosmcΔ256, and EDTA treated proteins. Cosmc was injected at 1.5 mg/ml onto a Sephacryl S300 size exclusion column (SEC) with phosphate buffered saline as the running buffer. SEC profiles are shown for untreated and EDTA treated **(A)** Cosmc WT; **(B)** CosmcE152K; **(C)** CosmcΔ256.

Next, we characterized the oligomeric behavior of Cosmc using size exclusion chromatography (SEC) (Fig 5). Cosmc WT, E152K, and Δ256 appear as a homogeneous species by SEC with a single peak. Using a set of protein standards (apoferritin, alcohol dehydrogenase, ovalbumin, carbonic anhydrase, RNaseA, vitamin B12) we created a calibration curve in order to estimate the molecular weight of Cosmc (S3 Fig). Using this method, the molecular weights of Cosmc and CosmcE152K were calculated to be 33 kDa, while for CosmcΔ256 it was 20 kDa. Unexpectedly, these values seem to be in agreement with the monomer weights for Cosmc and CosmcΔ256, which are 33.6 and 26.6 kDa, respectively.

Given the importance of the C-terminal domain in both oligomerization and metal binding, we hypothesized that metal binding may stabilize the oligomeric form of Cosmc. We characterized, therefore, the SEC profiles of Cosmc after EDTA treatment for comparison. Upon EDTA treatment, a species of smaller molecular weight appears for all Cosmc proteins tested, which corresponds to a calculated size of 7 kDa (Fig 5), which is likely the Cosmc monomer. It is noteworthy that in gel filtration the apparent molecular weight of Cosmc does not agree with its known molecular weight. This is probably due to unusual matrix effects, since when we used a different size exclusion column to characterize Cosmc (a TSKgelG2000SW), we observed the opposite result, in that Cosmc was heterogeneous and largely aggregated and eluted near the void volume. This anomalous behavior suggests that matrix interactions play a significant role in retention [32] (although we did not observe that the retention times change significantly with salt concentration). The smaller species for Cosmc, CosmcE152K, and CosmcΔ256 corresponds to a calculated molecular weight of 7 kDa, which is likely the monomeric species. CosmcΔ256 has an intermediate elution time, which is in agreement with our crosslinking studies that demonstrate this mutant exhibits defective oligomerization compared to WT.

## Discussion

### The N-terminal domain of Cosmc mediates client binding, while the C-terminal domain is necessary for oligomerization and metal binding

**Fig 6:**
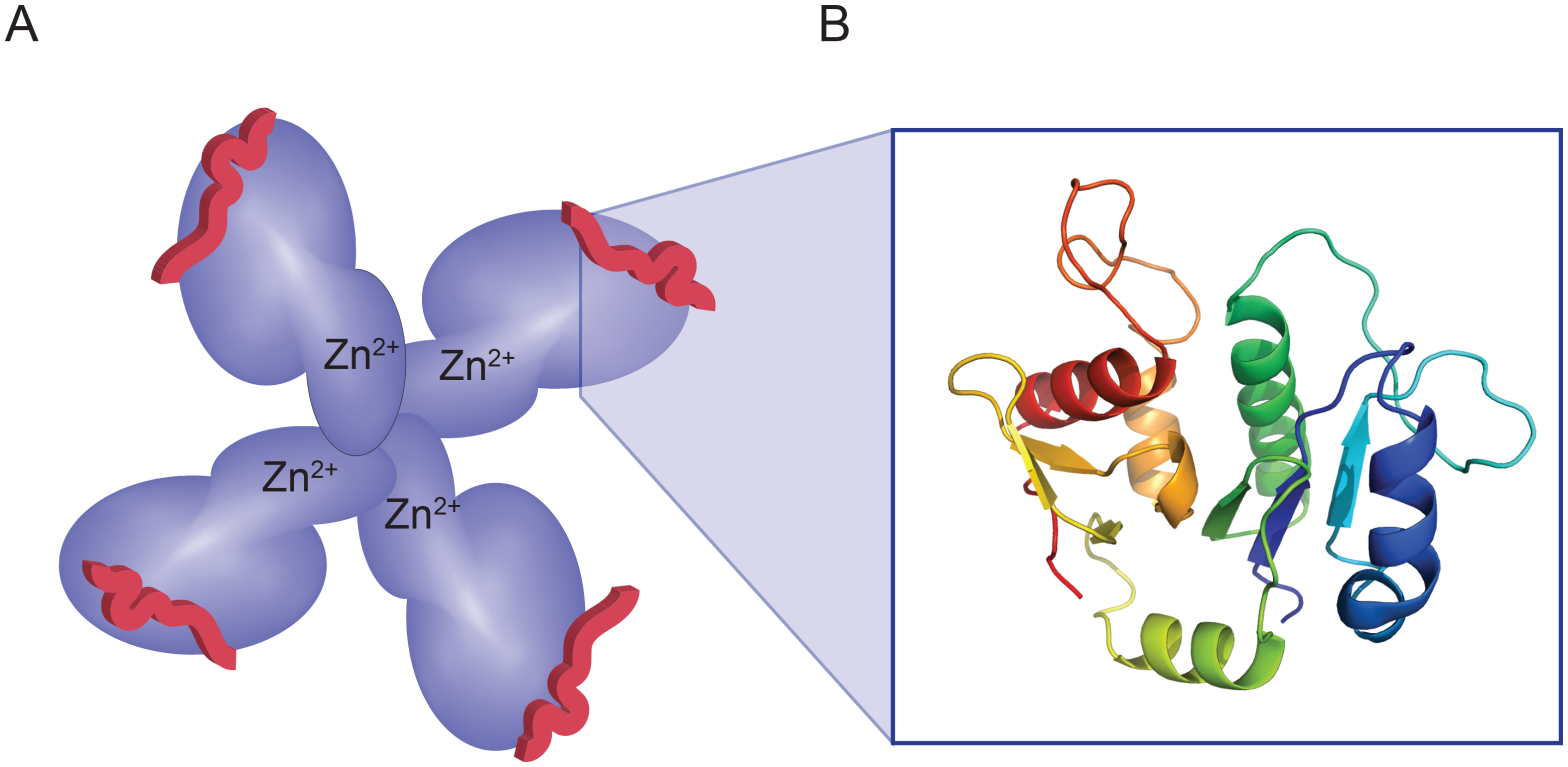
Cartoon model of Cosmc structure. **(A)** A representation of Cosmc with an N-terminal domain (dark blue) that is responsible for interacting with the client (red peptide) and a smaller, C-terminal domain (light blue) that mediates oligomerization and metal binding (Zn^2+^). A putative tetramer is shown for illustration purposes only, however our data is consistent with heterogeneous oligomeric states. **(B)** An atomic model of Cosmc’s glycosyltransferase homology region based on Mfng (PDB ID 2J0B) using SWISS-MODEL. The glycosyltransferase homology region within Cosmc is restricted to the N-terminal domain.

The major findings of our study are that the N- and C-terminal domains of Cosmc have very different functions. The N-terminal domain mediates *in vitro* chaperone function, and is directly involved in T-synthase binding, while the C-terminal domain mediates oligomerization and Zn^2+^ binding, which may serve a regulatory role *in vivo* (Fig 6A). Furthermore, our evidence suggests that heterogeneous oligomerization occurs at physiological concentrations, and that zinc is an endogenous ligand for Cosmc. These findings provide important new findings describing the structure of Cosmc, which provide new insight into its function as a unique molecular chaperone in regulating the folding of its client, T-synthase.

Cosmc is an essential protein in vertebrates and a key checkpoint of O-GalNAc glycosylation due to the regulation of its specific client, T-synthase. Despite the correlation of aberrant O-GalNAc type glycosylation with human disease and essential nature of both Cosmc and T-synthase, many basic biochemical properties have yet to be described for Cosmc. Properties including protein fold, domain structure, oligomerization behavior, and cofactor binding, provide valuable information towards understanding protein function.

The secondary structure of Cosmc is consistent with a GT-A folded protein, and due to its evolutionary relationship with the CAZy GT31 family of glycosyltransferases, it is possible that it also shares the conserved fold. Interestingly, GT folded proteins show low sequence identity 12% despite the shared fold [33]. Although Cosmc may adopt a GT-A fold, it deviates from the GT family in other biochemical features. For example, the cation specificity between Cosmc and GTs differs: T-synthase like many GT-A proteins requires Mn^2+^, while Cosmc binds Zn^2+^ [5, 18]. More importantly, the GT homology region does not mediate metal binding, but rather the C-terminus of Cosmc plays a role in cation interaction (Fig 6A and 6B). This observation is in line with the fact that Cosmc lacks a DxD motif, which coordinates divalent cation and sugar nucleotide substrate in GT structures [34]. Although the C-terminus is necessary for metal binding, it is not sufficient, as CosmcE152K, which lies within the N-terminal domain shows reduced metal binding. The negatively charged carboxylate of the Glu-153 sidechain may be positioned near the C-terminal domain such that it contributes a metal-bridged interaction.

Many GTs are oligomeric, however, oligomeric structures are much less conserved (compared to the GT fold). GTs may dimerize through a number of determinants, including the transmembrane and/or stem regions, where disulfide mediated linkages are common, directly through the GT domain, or via alternative epitopes [33]. T-synthase can be crosslinked into a homogeneous dimeric species, and the dimer correlates with enzyme activity, suggesting that T-synthase may be an obligate dimer [16, 19]. In contrast, the Cosmc monomer shows chaperone activity, and Cosmc heterogeneously oligomerizes in solution and in cell lysates, which may suggest a dynamic role of oligomerization in chaperone function and a possible point of *in vivo* regulation.

### Oligomerization and metal binding may be important regulators of Cosmc *in vivo*

More than half of the non-redundant protein structures in the Protein Data Bank are oligomeric, and oligomerization can be a direct modulator of function [35]. For example, the oligomerization state of G protein-coupled receptors directly modulates their signaling function [36]. For the chaperones Hsp90 and Hsp70, different oligomeric forms have different levels of chaperone function, and higher order Hsp90 oligomers are more active chaperones, while for Hsp70, the inverse is true–the Hsp70 monomer is the active chaperone species [37, 38]. Therefore, it has been suggested that oligomerization of these chaperones regulates the concentration of active chaperone in the cell, although the exact mechanisms are unclear. Our data suggest that oligomerization does not affect Cosmc chaperone activity, at least *in vitro*, and results from *in vivo* experiments may help elucidate the function of Cosmc oligomers. However, we note that the heterogeneity we observe at physiological condition may suggest that small perturbations can effectively shift the equilibrium.

Another important feature revealed in our studies is that the C-terminal domain of Cosmc mediates Zn^2+^ binding (Fig 6). Protein-metal interactions are common in protein biology and metal ions may serve to stabilize a protein fold, as in zinc finger proteins [39]; participate in catalysis or ligand binding, as in GTs [34]; have a regulatory role, as in the signaling molecule calmodulin [40]. In particular, Zn^2+^ and Ca^2+^ are important in regulating ER proteostasis, though the concentration of free Zn^2+^ is particularly low and is estimated at <1 pM in the secretory pathway [41]. Despite the tight regulation of cellular Zn^2+^, we found that overexpressed Cosmc purifies as a zinc-metalloprotein suggesting that endogenous Cosmc may be similarly bound to Zn^2+^. In the ER, Zn^2+^ modulates calreticulin and calnexin activity, and Zn^2+^ binding induces co-chaperone binding, and increases chaperone activity [42–44]. In our studies on Cosmc, we found no difference in *in vitro* function upon metal binding.

Both Cosmc and T-synthase share a conserved dicysteine (CC) motif near the C-terminus, and we suggest that it may have a regulatory role. CC motifs can function as redox sensors, and conformational change accompanies reduction of the disulfide bond, as adjacent cystines have a strained backbone conformation [45–49]. Additionally, reduced thiols often coordinate Zn^2+^, thus linking environmental redox conditions to metal binding function [50]. However, our results with Cosmc show that that thiol residues do not mediate metal binding or chaperone function. Our earlier studies on Cosmc indicated at least one out of 6 total cysteine residues present in the recombinant Cosmc construct was accessible to an alkylating agent as detected by a MALDI-MS mass shift of the intact protein, which is consistent with presence of an easily oxidizable disulfide bond [19].

### CosmcE152K is functional *in vitro* but not *in vivo*, highlighting the complexity of biological systems

CosmcE152K shows loss of function *in vivo*, but we unexpectedly found that it has WT activity *in vitro*. These results suggest caution in the interpretation and comparison of *in vivo* and *in vitro* mutational data. Prior work in our lab demonstrated a direct interaction between Cosmc and T-synthase in the *in vitro* chaperone assay, and therefore, defects in *in vitro* function are likely to be reflective of impaired client binding, although the assay may not precisely replicate the cellular chaperone pathway. We suggest that E152K does not interfere with T-synthase binding, but rather perturbs another feature of Cosmc biochemistry. In our studies, we found WT-like biochemical properties, with the exception of reduced Zn^2+^ binding activity for CosmcE152K, which will be addressed in future studies.

## Conclusions

In chaperone systems, chaperone cycling is often coupled to conformational switching between client-binding (open) and client-release (closed) states. While ATP binding and hydrolysis typically drives conformational cycling, alternatives including pH (for HdeA and Hsp47) and redox (for Hsp33) have been identified to promote client release [51, 52]. Another alternative chaperone mechanism in which the chaperone binds to native and nonnative states similarly has been described for Spy, a bacterial chaperone [53]. Thus these examples serve to illustrate that novel mechanisms are being discovered that may broaden the classical definition of chaperone-client interactions. Although the mechanisms are unclear, the GT family member POFUT1, was reported to show chaperone activity towards a specific client [54, 55]. Accumulating evidence suggests that client-restricted chaperones, like Cosmc, FACT (chromatin chaperone), TRiC/CCT (actin and tubulin chaperonin), and Hsp47 play key and nonredundant roles in biological systems [56–58]. For Cosmc/T-synthase, their specificity may arise from their unique shared evolutionary history, and studying their co-evolution may inform the mechanism of Cosmc function and elucidate the requirement of Cosmc for vertebrate T-synthase function.

Our results have important implications to understanding the mechanism of Cosmc function and provide novel structure-function data for Cosmc. Cosmc is composed of two, relatively independent domains: an N-terminal domain that mediates client binding, and a smaller, C-terminal domain that mediates oligomerization and Zn^2+^ binding. Our studies on the loss of function mutant, CosmcE152K, show that it supports client activity *in vitro*, though it does not function in the complex cellular milieu, and therefore this mutant is an important tool to elucidate the requirements for chaperone function *in vivo*. Cosmc oligomerization is significantly heterogeneous and may serve an important purpose, along with Zn^2+^ binding, which is a known regulator of ER proteostasis. The findings here describe novel biochemical features of Cosmc, which provide a basis to understand molecular mechanisms of the chaperone-client cycle for this essential pair.

## Materials and methods

### Cloning, protein expression and purification

Recombinant T-synthase was expressed and purified as described and used for *in vitro* chaperone assays [17]. Cosmc protein purified from insect cell hosts was also as described [17]. For Cosmc expression in mammalian cells, the soluble domain of Cosmc (residues 29–318 from the full length protein) was cloned into pGEN2 directly behind the signal sequence from the *Trypanosoma cruzi* α-mannosidase (MRLLTALFAYFIVALILAFSVSAKS) [59]. Point mutations were then made following Quikchange mutagenesis (Stratagene) and plasmids were verified by sequencing. Freestyle 293-F cells (Invitrogen), a suspension and serum-free adapted HEK293 cell line was transfected using polyethyleneimine. After 5–7 days, protein was purified from the culture supernatant by nickel affinity chromatography relying on the endogenous histidine rich region of Cosmc. Protein was stored at -80°C until use.

For bacterial expression, the *Escherichia coli* codon optimized *Cosmc* gene (encoding residues 29–318) was cloned into pET22b without exogenous tags. BL21Star ^**™**^ (DE3) (Life Technologies) cells were grown at 37°C in Luria-Bertani medium, and protein expression was induced with isopropyl β-D-1-thiogalactopyranoside (IPTG) at mid-log phase (A_600_ = 0.6) for 3–4 hrs. Cells were harvested by centrifugation, resuspended (10 mM Tris, pH 8.0, with 1 mM MgCl_2_) and frozen at -80°C. Cells were lysed by sonication, and inclusion bodies containing Cosmc were collected by centrifugation, and washed twice with 10 mM Tris, pH 8.0, 0.1% TritonX-100, and once with 10mM Tris pH 8.0, 2M NaCl. The subsequent pellet was dissolved in denaturant (6M guanidinium chloride, 20 mM Tris, pH 8.0) and Cosmc was purified using nickel affinity chromatography, relying on the endogenous histidine rich region to support purification (Qiagen). Cosmc was refolded by dilution into renaturing buffer (50 mM Tris, pH 8.2, 250 mM NaCl, 10 mM KCl, 300 mM L-Arginine, 300 mM sucrose, 8 mM reduced glutathione, 3 mM oxidized glutathione) for 2 hours at room temperature before being dialyzed into storage buffer and frozen at -80°C until use.

### Limited proteolysis

Cosmc was subjected to limited trypsinolysis at a molar ratio of 1:500 (trypsin:Cosmc) and 0.5mg/ml of Cosmc in 20mM Tris, 250mM NaCl, pH 7.4. Aliquots were removed over a 3-hour time course and quenched by heating in SDS-PAGE sample buffer. Protein products were analyzed by SDS-PAGE, Coomassie staining, and mass spectrometry sequencing at the Emory University Proteomics Core.

### *In vitro* chaperone assays

WT or mutant protein was thawed and dialyzed overnight into 20 mM Tris, pH 7.5, 250 mM NaCl in preparation for functional assays. For the EDTA treated experiments, WT protein was thawed, and treated with 1mM EDTA on ice for 30 min and then dialyzed against the buffer above. The alkylated protein was prepared by treating Cosmc with 5 mM β-mercaptoethanol followed by 50 mM iodoacetamide and subsequent dialysis overnight against the buffer above. *In vitro* chaperone assays were performed by adding 2 uM, 600 nM, or 200 nM Cosmc to recombinantly purified T-synthase protein preparations in reaction buffer (10 mM HEPES, 10 mM MgCl2, and 150 mM NaCl pH 7.8) at room temperature for 30 minutes. T-synthase activity was measured as described [60]. Chaperone activity was calculated as Signal_(chaperone reaction)_ − Signal_(background)_, where the chaperone reaction consists of Cosmc, T-synthase and T-synthase substrates, and the background reaction consists of Cosmc and T-synthase substrates. The activity of T-synthase alone is shown as a separate control. Assays were performed in quadruplicate and the average and standard deviations are reported.

### Circular dichroism spectroscopy and thermal melts

Circular dichroism (CD) spectra were recorded on a J-810 spectropolarimeter (Jasco) at 25°C from 260 to 190 nm with a bandwidth of 2 nm. For CD experiments, proteins were dialyzed overnight into 10 mM potassium phosphate, 250 sodium fluoride at pH 7.4 and spectra were recorded at a protein concentration of 0.1 mg/ml in a 1mm pathlength cuvette. CD signal at 222 nm was collected from 20°C–80°C for Cosmc and CosmcE152K, and from 20°C–95°C for CosmcΔ256 every 0.5°C with a slope of 1°C/min and bandwidth of 2.0 nm.

### Oligomerization studies

For chemical crosslinking, Cosmc, IgG (Equitech-Bio, Inc), and BSA (Sigma) proteins were dialyzed into 20 mM sodium phosphate pH 7.5, 250 mM NaCl. Crosslinking was conducted by addition of 0.5 mM disuccinimidyl glutarate (DSG, ThermoFisher) for 30 min at room temperature at a protein concentration of 0.3 mg/ml. Reactions were quenched with 1/10 volume of 100 mM Tris pH 7.5 for 30 min at room temperature and analyzed by SDS-PAGE and Coomassie G-250 staining. For blue native PAGE, proteins were adjusted to 0.3 mg/ml with 10 mM Tris pH 7.5, 50 mM NaCl, and sample buffer including Ponceau S and glycerol was added. BN-PAGE (Invitrogen) was run at 4°C with Coomassie G-250 dye in the Cathode buffer and stained with Coomassie G-250. For chemical crosslinking of full length Cosmc in mammalian cells lysates, we utilized HEK293 Simple Cells, HEK293SC (a kind gift of Henrik Clausen), which lack endogenous Cosmc. Full length Cosmc with a C-terminal HPC4 tag was cloned into pcDNA3.1 and stably transfected into HEK293SC. Cells were lysed in 20mM sodium phosphate, pH 7.5, 250 mM NaCl, 1% Triton-X100, 0.5% sodium deoxycholate containing protease inhibitors (Roche) with gentle sonication and clarified by centrifugation. Lysates were crosslinked with 5mM DSG or treated with vehicle control for 20 min at room temperature and quenched with 1/10 volume of 1M Tris pH 7.5 for 20 min at room temperature. SDS-PAGE samples were prepared and analyzed by western blot using an HPC4 antibody to detect Cosmc and a β-actin antibody as a negative control.

### Metal binding assays

Cosmc proteins were dialyzed into 20 mM Tris pH 7.4, 250 mM NaCl. For treatment with a cation chelator, 1mM EDTA was added and incubated at 4°C for 1 hr before dialysis into 20 mM Tris pH 7.4, 250 mM NaCl. Thermal shift assays were performed with 3 uM Cosmc protein and SYPRO Orange (Invitrogen) at a final concentration of 5x with 1mM divalent cation present. Fluorescence intensity measurements were recorded from 21°C to 95°C in 1.0°C steps, in 96-well plate format qPCR thermal cycler (Applied Biosystems). Data were fit to a Boltzman curve with sloping baselines with OriginPro (OriginLab) to locate the inflection point (T_m_). Assays were performed in triplicate and mean and standard deviation are reported. The analysis of trace metals in recombinant Cosmc was performed by the Harvard Trace Metal Lab at the Harvard T.H. Chan School of Public Health.

### Size exclusion chromatography

A HiPrep 26/60 Sephacryl S300 HR column (GE Healthcare) was used on a Shimadzu HPLC system for SEC studies. Phosphate buffered saline (PBS) was used as a running buffer at a flow rate of 1 ml/min. Cosmc proteins were dialyzed overnight into PBS, or treated with 10 mM EDTA and dialyzed into PBS containing 1mM EDTA overnight, and injected at a protein concentration of 1.5mg/ml. Absorbance was monitored at 220 nm and 280 nm. Apoferritin, alcohol dehydrogenase, ovalbumin, carbonic anhydrase, RNaseA, and vitamin B12 (Sigma) were used as protein standards to create a calibration curve (S3 Fig). Blue dextran was used to calculate the void volume of the column.

## Supporting information

S1 Fig PNGaseF treatment of Cosmc. After overnight treatment with PNGaseF (+) Cosmc does not show a gel shift, which is consistent with a non-N-glycosylated protein. RNaseB has a single N-glycosylation site and shows a gel shift after PNGaseF digestion, from 15.5 kDa to 13.7 kDa. In contrast, BSA, not N-glycosylated, does not produce a gel shift.
(TIF)

S2 Fig Limited proteolysis of Cosmc. Limited proteolysis of Cosmc purified from mammalian cells (HEK293F), bacterial cells (*E*. *coli* BL21DE3), and recombinant CosmcΔ256 purified from mammalian cells (HEK293F), all show similar patterns.
(TIF)

S3 Fig Cosmc size exclusion chromatography. The calibration line and data points for standard proteins (black diamonds) and Cosmc proteins (gray circles) are plotted as log[molecular weight} as a function of retention. The value for K_ave_ was calculated as K_ave_ = (V_ret_ − V_0_)/ (V_c_ − V_0_), where V_0_ is the void volume, experimentally determined with Dextran Blue (95.0 mL), and V_c_ is the calculated column volume, 320 mL, and V_ret_ is the retention volume for each protein from the chromatogram.
(TIF)

## Abbreviations

BN-PAGE: blue native PAGE
CD: circular dichroism
DSG: disuccinimidyl glutarate
ER: endoplasmic reticulum
GT: glycosyltransferase
*T_m_*: midpoint of the thermal unfolding transition
WT: wild type
