# Supplementary figures and images for: Biochemical characterization of functional domains of the chaperone Cosmc
Source: PLoS One. 2017 Jun 30;12(6):e0180242. doi: 10.1371/journal.pone.0180242 (PMC5493369; doi:10.1371/journal.pone.0180242)

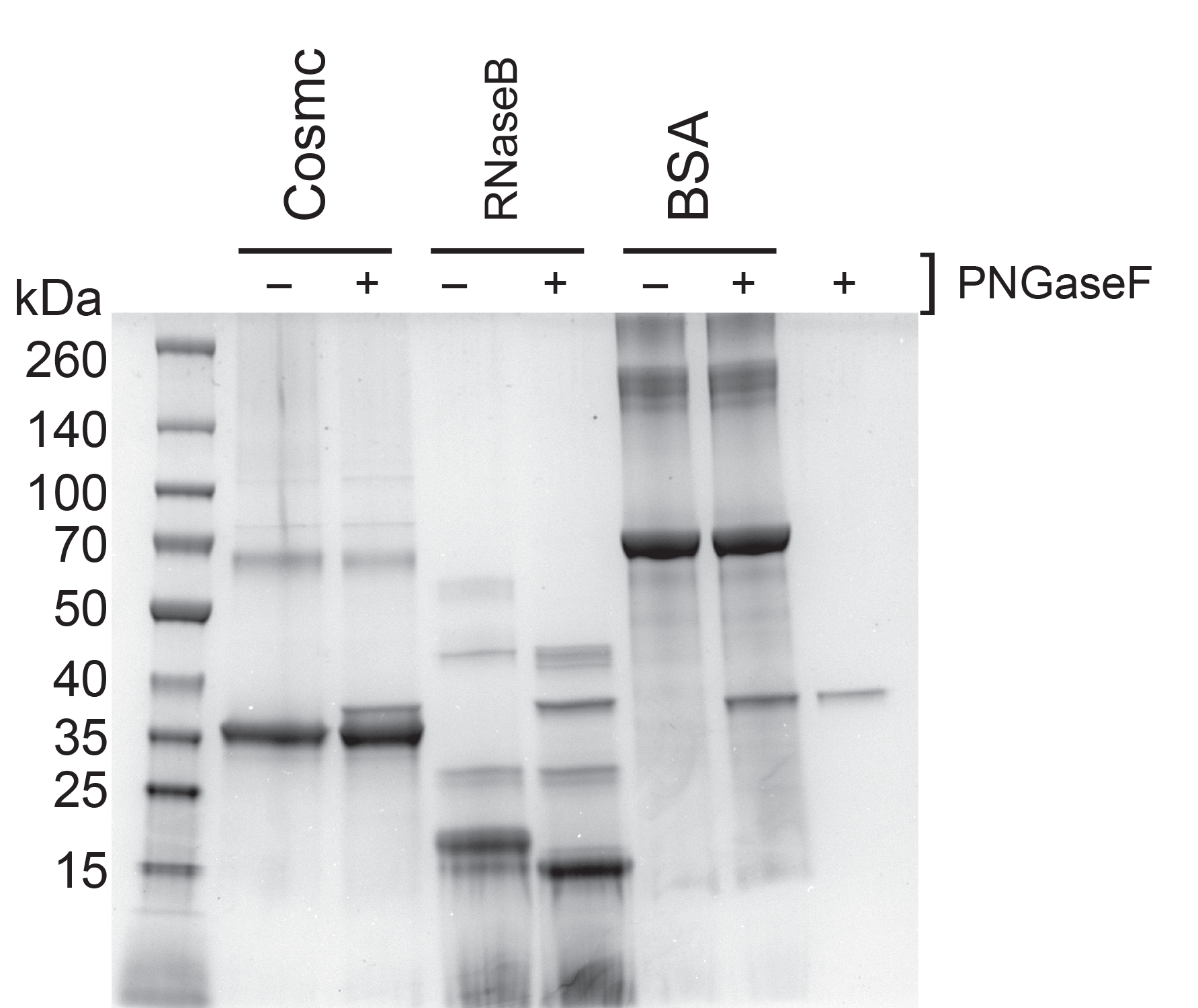

Supplement: S1 Fig — After overnight treatment with PNGaseF (+) Cosmc does not show a gel shift, which is consistent with a non-N-glycosylated protein. RNaseB has a single N-glycosylation site and shows a gel shift after PNGaseF digestion, from 15.5 kDa to 13.7 kDa. In contrast, BSA, not N-glycosylated, does not produce a gel shift. (TIF) [file pone.0180242.s001.tif]

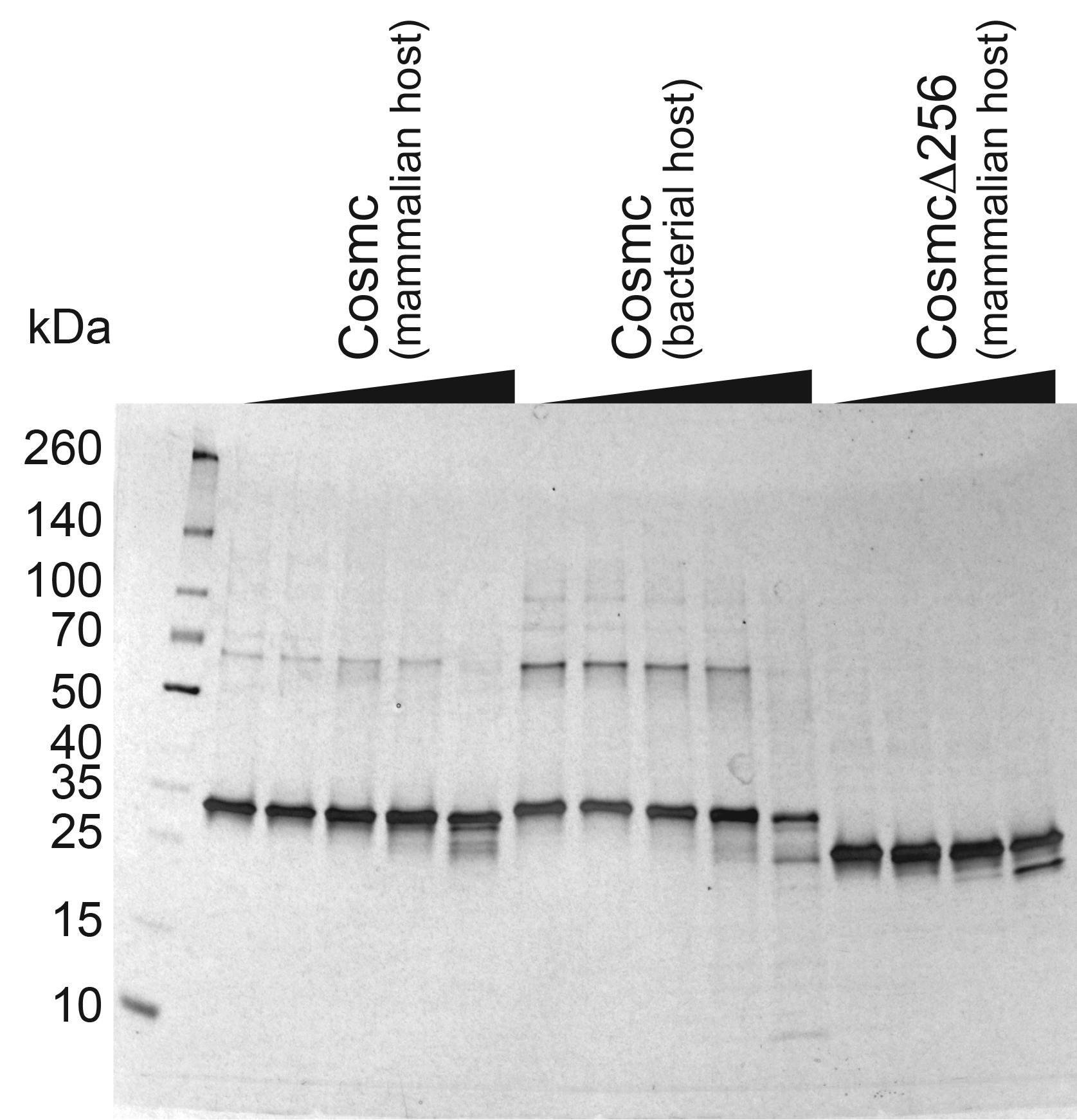

Supplement: S2 Fig — Limited proteolysis of Cosmc purified from mammalian cells (HEK293F), bacterial cells (E. coli BL21DE3), and recombinant CosmcΔ256 purified from mammalian cells (HEK293F), all show similar patterns. (TIF) [file pone.0180242.s002.tif]

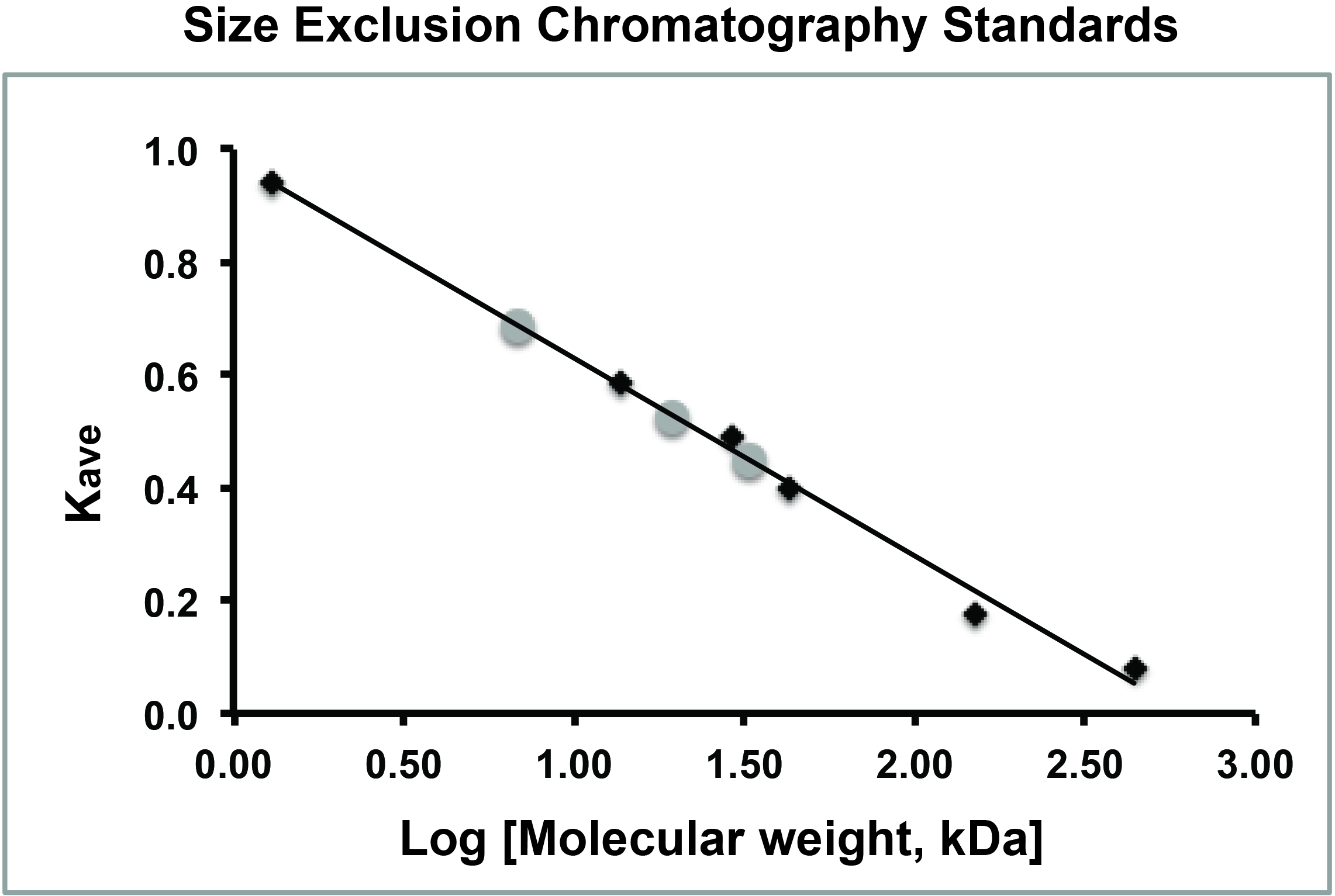

Supplement: S3 Fig — The calibration line and data points for standard proteins (black diamonds) and Cosmc proteins (gray circles) are plotted as log[molecular weight} as a function of retention. The value for Kave was calculated as Kave = (Vret − V0)/ (Vc − V0), where V0 is the void volume, experimentally determined with Dextran Blue (95.0 mL), and Vc is the calculated column volume, 320 mL, and Vret is the retention volume for each protein from the chromatogram. (TIF) [file pone.0180242.s003.tif]
